# Resistance management and integrated pest management insights from deployment of a Cry3Bb1+ Gpp34Ab1/Tpp35Ab1 pyramid in a resistant western corn rootworm landscape

**DOI:** 10.1371/journal.pone.0299483

**Published:** 2024-03-08

**Authors:** Lance J. Meinke, Jordan D. Reinders, Timothy B. Dang, Jeffrey T. Krumm, Clinton D. Pilcher, Matthew W. Carroll, Graham P. Head

**Affiliations:** 1 Department of Entomology, University of Nebraska, Lincoln, Nebraska, United States of America; 2 Midwest Research, Hastings, NE, United States of America; 3 Corteva Agriscience, Johnston, IA, United States of America; 4 CropScience Division, Bayer AG, Chesterfield, MO, United States of America; Assam Agricultural University Faculty of Agriculture, INDIA

## Abstract

In Nebraska USA, many populations of western corn rootworm (WCR), *Diabrotica virgifera virgifera* LeConte, now exhibit some level of resistance to all corn rootworm-active *Bacillus thuringiensis* Berliner (Bt) proteins expressed in commercial hybrids. Therefore, a study was conducted in northeast Nebraska from 2020–2022 to reevaluate current corn rootworm management options in continuous maize (consecutive planting for ≥2 years). Results from on-farm experiments to evaluate a standard soil-applied insecticide (Aztec® 4.67G) in combination with non-rootworm Bt or rootworm-active Bt pyramided maize (Cry3Bb1 + Gpp34Ab1/Tpp35Ab1) are reported within the context of WCR Bt resistance levels present. Corrected survival from Bt pyramid single-plant bioassays (<0.3, 0.3–0.49, >0.5) was used to place populations into 3 resistance categories. Variables evaluated included root injury, adult emergence, proportion lodged maize, and grain yield. Key results: A composite analysis of all populations across resistance levels indicated that addition of soil insecticide to Bt pyramid significantly reduced adult emergence and lodging but did not significantly increase root protection or yield. Within and among resistance category analyses of root injury revealed that the Bt pyramid remained highly efficacious at any non-rootworm Bt root injury level when resistance was absent or low. When corrected survival was >0.3, mean Bt pyramid root injury tracked more closely in a positive linear fashion with mean non-rootworm Bt root injury (rootworm density x level of resistance interaction). Similar trends were obtained for adult emergence but not yield. Mean Bt pyramid root injury rating was <0.75 in most populations with Bt resistance, which contributed to no significant yield differences among categories. Results are discussed within the context of IPM:IRM tradeoffs and the need to reduce WCR densities in this system to decrease the impact of the density x resistance interaction to bridge use of current pyramids with new technologies introduced over the next decade.

## Introduction

The western corn rootworm (WCR), *Diabrotica virgifera virgifera* LeConte, is a univoltine Chrysomelid beetle species which was first documented feeding on maize (*Zea mays* L.) roots in Colorado USA in 1909 and became an invasive pest species in southwest Nebraska during the 1920s [1–3]. The WCR feeds on roots of some grasses with maize being an optimal host [4–9]. Annual rotation from maize to a crop that would not support WCR larval survival was the recommended WCR management tactic as early as 1930 in southwestern Nebraska, but the profitability of maize led some growers to start planting continuous maize (maize planted for ≥2 years in one location) [2,3]. By the 1940s, WCR injury to continuous maize in central Nebraska was common [10,11] and the species subsequently increased its geographic range across the United States (U.S.) Corn Belt to New Jersey by the 1980s [12,13]. Today, continuous maize is still a profitable agronomic practice to support the annual demand for maize as feed for the large confined livestock industry and ethanol production. This agricultural system provides consistent habitat that can facilitate build-up of WCR densities and increase larval injury over time [13,14], which has elevated the WCR to key pest status across the U.S. Corn Belt [12]. Larval injury to maize roots can cause plant instability, reduced plant growth, and significant yield loss [15–21]. Yield losses caused by WCR feeding injury and associated control costs can exceed $2 billion USD annually [22].

The corn rootworm transgenic era began in the early 2000s when three rootworm-active plant-incorporated proteins derived from the soil bacterium *Bacillus thuringiensis* Berliner (Bt) were marketed as single-protein hybrids: Cry3Bb1 in 2003 [23]), Gpp34Ab1/Tpp35Ab1 (original taxonomy: Cry34/35Ab1 [24]) in 2005 [25], and mCry3A in 2006 [26]. Transgenic maize has largely replaced soil and foliar insecticides as the primary WCR management tactic in continuous maize [27] due to increased efficacy and ease of use [28]. However, none of the commercially available Bt proteins targeting the WCR are expressed at high-dose levels [29–34] (i.e., dose required to kill >99% of homozygous susceptible insects that produces 25 times more toxin than is required to kill susceptible individuals [35,36]), which has contributed to rapid selection for field-evolved resistance in areas of the leading U.S. maize-producing states [37–50]. Bt resistance has evolved after consecutive use of the same Bt protein for as little as 3 years in the field [37,40]. A positive correlation exists between the number of consecutive years Bt maize is grown and survival of WCR populations in Bt maize laboratory bioassays [37,39,46].

To delay WCR resistance evolution or mitigate single-protein resistance, corn hybrids expressing two or more rootworm-active Bt proteins, defined as ‘pyramids’ [47,51], were registered and have replaced single protein hybrids in the marketplace [36,47]. Most pyramids were developed through cross-licensing agreements among rootworm-trait registrants (exception: mCry3A + eCry3.1Ab). All current pyramids contain one or more proteins that were originally sold as single-protein products. Previous exposure of WCR populations to individual proteins conferring various levels of single-protein resistance can potentially compromise current commercial pyramids [39,47,49,52]. Variable levels of cross-resistance among the three registered Cry3 proteins have been documented but none show cross-resistance with Gpp34Ab1/Tpp35Ab1 [37,38,40,42,43]. Therefore, the Gpp34Ab1/Tpp35Ab1 protein has been paired with a Cry3 protein in commonly planted pyramids [50]. A recently commercialized pyramid includes a novel RNAi trait (DvSnf7 dsRNA) but also includes both Cry3Bb1 and Gpp34Ab1/Tpp35Ab1 [53]. So currently, it is not possible to rotate rootworm-active Bt proteins as an effective insect resistant management (IRM) strategy to mitigate Bt resistance evolution [50].

Extending the durability of current Bt hybrids is paramount to bridge the transition to new control tactics that may be available in the next decade. There is a need to integrate transgenic maize with existing integrated pest management (IPM) tactics to develop economical and effective IRM programs that delay or mitigate resistance. Therefore, a project was conducted during 2020–2022 in northeast Nebraska to evaluate existing WCR management practices, (i.e., rotation to a non-host crop [2,54]; use of soil- or foliar-applied insecticides to complement non-rootworm Bt or rootworm-active Bt pyramided maize [27]) for potential to reduce WCR densities and associated larval injury in continuous maize and contribute to mitigation of WCR Bt resistance. Results from on-farm experiments to evaluate a soil-applied insecticide in combination with non-rootworm Bt or rootworm-active Bt pyramided maize are reported in this paper within the context of WCR Bt resistance levels present. The study was conducted in northeast Nebraska because the landscape contains a large number of continuous maize fields (2 to > 10 years) associated with a high concentration of confined livestock. In addition, long-term use of Cry3, Gpp34Ab1/Tpp35Ab1 and Bt pyramided maize hybrids containing Gpp34Ab1/Tpp35Ab1 to manage WCR injury is common in the area and WCR populations exhibit various levels of resistance to Cry3 and Gpp34Ab1/Tpp35Ab1 proteins [40,47,50,55]. The objectives addressed in this paper include: i) determine the impact of a non-rootworm Bt hybrid and the Cry3Bb1 + Gpp34Ab1/Tpp35Ab1 pyramid (hereafter referred to as ‘non-RW Bt’, ‘Bt pyramid’, respectively, throughout the manuscript) with and without a standard granular soil insecticide on the variables root injury, adult emergence, proportion lodged maize, and grain yield; ii) evaluate the effect of WCR Bt pyramid resistance levels on pyramid root injury, adult emergence, and yield.

## Materials and methods

### WCR populations/fields

**Fig 1:**
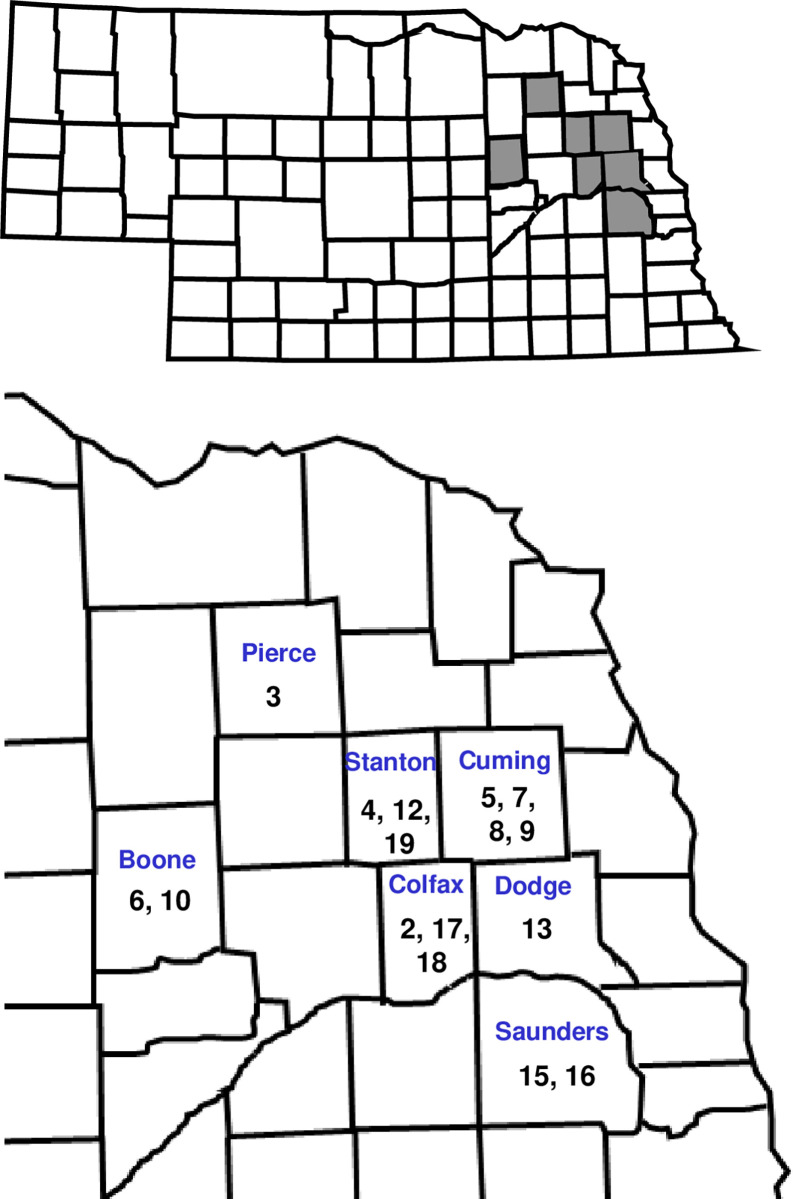
Nebraska state map showing counties in gray where on-farm research was conducted. The expanded map of northeast Nebraska includes the unique numbers assigned to each field/WCR population used in the project.

Farmer cooperators were identified in seven counties in northeast Nebraska. Cooperators provided permission to conduct this project on their farms. A unique number was assigned to each field/WCR population used in the project (Fig 1). The majority of fields were selected for the study based on the following criteria: planted to continuous irrigated maize for at least 2 years (many 5- >10 years), history of use of rootworm-active single-protein and Bt pyramid hybrids, moderate-high WCR densities present, significant risk from WCR injury and Bt resistance possible. A small number of fields were selected from within the landscape that had little to no past exposure to rootworm-Bt proteins. These included commercial field 13, and fields 15 and 16 which were continuous maize located about 4km apart on the University of Nebraska Eastern Nebraska Research, Extension, and Education Center in Saunders Co. General background of each field is presented in Table 1.

**Table 1:** General background: Fields where strip trials were planted.

Field Number	Strip-Trial Years	Field Size (Ha)	General Agronomic/Trait History, 2018–2022
2	2020–2022	56.7	Mix: Cry3 many years prior to non-RW Bt + soil insecticide, 2019–2022
3	2020–2022	32.4	Pyramid
4	2022	39.3	Pyramid, soybean in 2020
5	2020	29.1	Pyramid
6	2020–2021	60.9	Pyramid: 2018–2020
7	2020–2022	13.4	Pyramid
8	2020–2022	29.3	Pyramid
9	2020–2022	11.8	Pyramid
10	2020–2022	99.0	Pyramid, except 2019: Gpp34Ab1/Tpp35Ab1
12	2020–2022	60.7	Pyramid
13	2020	121.4	Conventional hybrid + soil insecticide for many years prior to pyramid in 2020
15	2020–2022	4.9	Minimal rootworm exposure to Bt traits
16	2020–2022	6.1	Minimal rootworm exposure to Bt traits
17	2021	56.7	Cry3 use prior to 2019; soybean in 2019, non-RW Bt + soil insecticide: 2020–2021
18	2022	85	Cry3 use prior to 2019; part pyramid/soybean in 2018–2020, non-RW Bt + soil insecticide, 2021–2022
19	2022	26.3	Pyramid 2018, 2019, 2022, soybean in 2020, non-RW + soil insecticide 2021

^1^Pyramid = mCry3A or Cry3Bb1 + Gpp34Ab1/Tpp35Ab1, non-RW Bt = no expression of corn rootworm-active Bt traits.

### On-farm experiment: Field design/data collection

Each field was an experimental unit or replicate. To provide consistent comparisons across fields, a Bt pyramid hybrid and a hybrid of similar genetic background with Lepidoptera-active traits but without rootworm-active traits (non-RW Bt) were planted in each field at 79,074 plants per ha (32,000 plants/A) to evaluate corn rootworm root injury, adult emergence, proportion lodged plants, and crop yield. Treatments included: 1) non-RW Bt hybrid + Aztec® 4.67G (primarily tebupirimphos + cyfluthrin) (AMVAC® Chemical Corporation, Newport Beach, CA) @ 85 gm/305 m (3 oz/1000 ft) (SAI hereafter), 2) non-RW Bt hybrid, 3) Bt pyramid hybrid + SAI; and 4) Bt pyramid hybrid. Each treatment was a 4-row (3.1 m) x ca. 61.5 m in length and all seed was treated with clothianidin at 0.5 mg/seed. During 2020, 2021, and 2022, this design was placed in 11, 11, and 12 continuous maize fields, respectively (Table 1). Fields were managed using commercial practices appropriate for the region with farmers applying fertilizer and herbicides to plots as part of applications to the entire field each strip trial was placed in. Plots were kept weed-free.

#### Adult emergence

Individual plant cages were placed over four total plants in one of the center two rows of each treatment. Each cage was ca. 12 m apart. Emergence cages were a modification of the design described by Fisher [56] but allowed the caged plant to remain intact and grow up through the center of the cage [57]. Emerging WCR adults were collected in a glass jar that included an inverted paper Konie cup with tip cut off (Konie Cups International Inc., Miami, FL). Jars were changed weekly and the total number of WCR in each jar was counted.

#### Root injury

During late July each year, individual plants were dug from the plant row that contained single-plant emergence cages to measure root injury. In total, 10 plants were randomly selected at ca. 4m intervals from each treatment. Roots were washed and injury rated using the 0–3 node injury scale (NIS, [58]). WCR egg density levels and associated NIS ratings are highly correlated [59] so the associated general larval pressure present (i.e., low, medium, high) can be inferred from NIS ratings. NIS ratings were used as an indirect measure of larval pressure or density in this study.

#### Proportion lodged plants and grain yield

Prior to harvest in each treatment, 22.9 row-m (75 row-ft) was measured in the center plot row adjacent to the row containing emergence cages. The total number of plants and the number of lodged plants (leaning ≥ 45° from vertical) were counted and the proportion of lodged plants in each treatment was calculated. All ears from the measured 22.9m were hand-harvested during late September-October when crop phenology had reached maturity. Ears were placed in mesh bags and returned to a greenhouse at the University of Nebraska-Lincoln to dry down to approximately 10–12% moisture. Corn ears from each treatment were shelled using a small batch sheller (ALMACO Maizer, Nevada, IA). Total grain weight and percentage moisture were determined from each treatment. Yield was converted to 15.5% moisture for each plot prior to statistical analysis.

### WCR single plant bioassays

#### WCR populations

Adult WCR were annually collected from each field included in the on-farm experiment during August 2019–2021. A minimum of 50 gravid females (usually >150) were collected from each field near the location where on-farm strip trials would be placed the following year to obtain a subset of the natural variation present. Field-collected adult WCR were transported to the Department of Entomology at the University of Nebraska-Lincoln and maintained by population in 28cm^3^ plexiglass cages under laboratory conditions during the summer and fall of each year. About 10,000 eggs were obtained per population each year. The procedural steps used to maintain adults, collect eggs, and the temperature regimens used to facilitate egg diapause and post-diapause development are described in Wangila et al. [40].

Diapausing WCR colonies reared and maintained at the USDA-ARS North Central Agricultural Research Laboratory in Brookings, South Dakota, were used as lab control populations (LAB-S). Each control population was collected prior to the initial commercialization of Bt proteins in 2003 and has been continuously reared without the addition of wild-type genes, preserving susceptibility to rootworm-active transgenic maize. Populations originated from collections in Butler County, Nebraska (1990), Potter County, South Dakota (1995), Finney County, Kansas (2000), and Centre County, Pennsylvania (2000).

#### Bioassay procedure

Neonate progeny of the F_1_ generation from each population were used in bioassays as described by Gassmann et al. [37] and adapted by Wangila et al. [40] and Reinders et al. [46]. This standardized technique is used to detect small shifts in WCR susceptibility to Bt proteins. Bioassays were conducted during the spring to summer of the year following beetle collection after termination of obligatory egg diapause (e.g., 2020 bioassays conducted with progeny of 2019 field collections). Two sets of bioassays were conducted simultaneously with hybrids of different genetic backgrounds. The first set included three maize hybrids without seed treatments: single-protein Cry3Bb1, the Bt pyramid, or no rootworm-Bt traits. The second set included two maize hybrids without seed treatments expressing Gpp34Ab1/Tpp35Ab1 or no rootworm-Bt traits. The same hybrids were used for all bioassays conducted during 2020–2022. Twelve plants of each hybrid were grown in individual 1L plastic pots (Johnson Paper & Supply Co., Minneapolis, MN) until the V4-V5 growth stage [60] to assay each WCR population. Twelve randomly selected F_1_ neonate larvae (≤24h after eclosion) were then placed on the roots of each individual plant and pots were held at 24°C with a 14:10 (L:D) photoperiod for 17 days. Each plant and surrounding soil was then placed in a separate Berlese funnel (40 W, 120 V lightbulbs) for 4 days to extract larval survivors. Seed was provided by Bayer CropScience (Cry3Bb1, Cry3Bb1 + Gpp34Ab1/Tpp35Ab1, no rootworm trait near isoline) and Corteva Agriscience (Gpp34Ab1/Tpp35Ab1, no rootworm trait near isoline) for use in bioassays.

### Data analysis

All data were analyzed using SAS 9.4 software [61]. Statistical significance was reported at α = 0.05 for all analyses. Fields 5 (2020), 4 (2022), and 12 (2022) were excluded from all yield analyses because severe moisture stress (unable to irrigate) in the latter half of the respective growing seasons greatly reduced yield to atypical levels compared to irrigated maize (S1–S3 Tables). Results from LSMEANS and associated standard errors are reported in this manuscript.

#### Bioassay corrected survival

Bioassay proportional survival was calculated on a per plant basis by dividing the number of larval survivors by 12 (i.e., number of larvae infested per plant). Corrected survival on the Bt pyramid hybrid and each single Bt protein was calculated as survival on each Bt bioassay plant divided by mean survival on the non-RW Bt hybrid for each population [62]. A linear model (implemented using PROC GLIMMIX^,^ [61]) following a normal distribution with unequal variances between populations was used to evaluate corrected survival [39,47] within each Bt hybrid separately for each year assays were conducted (2020–2022). WCR population was included in the model as a fixed factor. Normality assumptions and model fit were assessed by examining residual plots and heterogenous variance between populations was allowed to control for nonconstant variance by specifying GROUP = Population in the random statement. The DIFFS option was used to identify significant differences in corrected survival among WCR populations within each Bt hybrid. Data from LAB-S populations were pooled within hybrids and year to create a composite sample as initial analyses indicated no significant difference in survival among LAB-S populations on Cry3Bb1, Gpp34Ab1/Tpp35Ab1, or Bt pyramid maize (S4–S6 Tables).

Bt pyramid corrected survival from 2020 and 2022 of populations 3, 7–10, and 12 was also compared to estimate if susceptibility had changed during the duration of the project. These populations were selected for analysis as locations were continuously planted to maize containing Cry3 or Gpp34Ab1/Tpp35Ab1 single and more recent pyramid Bt traits for at least 10 consecutive years. A generalized linear model (implemented using PROC GLIMMIX [61]) with a one-way treatment structure was used to analyze the effect of strip trial year on WCR corrected survival. Year was included in the model as a fixed factor. The LSMEANS statement with the PDIFF option was used to identify significant differences in WCR corrected survival between strip trial years.

#### On-farm experiment: Overall analysis

Fields and associated WCR populations from each year the experiment was conducted (2020–2022) were included in an analysis to obtain a composite landscape view of total emergence, root damage, plant lodging, and yield across varying Bt susceptibility levels in northeast Nebraska. A generalized linear mixed model (GLMM; GLIMMIX procedure [61]) was used to analyze the effect of soil insecticide*hybrid treatments on total WCR emergence (negative binomial with a log link function), root damage rating (proportion; average of 10 plants following a normal distribution by Central Limit Theorem, CLT), proportion of lodged plants (beta distribution), and yield (normal distribution). Soil insecticide, hybrid, year, and all interactions were treated as fixed effects. Initial results indicated that the main effect of year and all interactions including year were not significant in any analysis so year was dropped from the model. The final model for each variable included hybrid, insecticide, and hybrid*insecticide as fixed effects and field*year was included in the model as a random factor to account for an overdispersion of variance. The LSMEANS statement with the SLICE option was used to identify significant differences in strip trial variables among treatments.

### On-farm experiment: Effect of WCR Bt resistance

*Influence of resistance level on non-RW Bt*:*Bt pyramid strip trial metrics*. WCR populations from 2020–2022 sites were pooled by susceptibility to the Bt pyramid into three corrected survival categories (low: <0.3, moderate: >0.3–0.49, high: ≥0.5 corrected survival). A generalized linear model (implemented using PROC GLIMMIX [61]) with a one-way treatment structure was used to analyze the effect of corrected survival category on the ratio of non-RW Bt:Bt pyramid strip trial metrics NIS, adult emergence, and yield. Corrected survival category was included in the model as a fixed factor. The LSMEANS statement with the PDIFF option was used to identify significant differences in non-RW Bt:Bt pyramid ratios among WCR corrected survival categories. Tukey’s multiplicity adjustment was used to control for type I error rates.

*Linear regressions*: *non-RW Bt x Bt pyramid for strip trial metrics*. A linear regression model (implemented using PROC GLIMMIX [61]) was used to determine the intercept and slope of the relationship between non-RW Bt and Bt pyramid strip trial metrics for mean NIS and total WCR emergence, respectively, plus mean NIS and yield for each corrected survival category (low, moderate, high, previously described) with the following model:

Bt pyramid strip trial metric = β0 + β1 · non-RW Bt strip trial metric + e

where β0 is the intercept, β1 is the slope associated with the WCR non-RW Bt metrics, and errors (denoted e) are assumed to be independent and normally distributed with a variance of σ^2^. Pearson’s correlation coefficient was used to measure the strength of association between non-RW Bt and Bt pyramid strip trial metrics for each corrected survival category using PROC CORR [61].

## Results

### Bioassay corrected survival

Significant variation in Bt pyramid corrected survival occurred among populations during each year of the experiment (2020: *F*_15,114.6_ = 22.83, *p* <0.0001; 2021: *F*_12,95.2_ = 55.37, *p* <0.0001; 2022: *F*_13,102.5_ = 37.28, *p* <0.0001; Tables 2–4). Corrected survival ranged from 0.00–0.68, 0.01–1.07, and 0.01–0.85 during 2020, 2021, and 2022, respectively. LAB-S corrected survival was very low each year. Except for populations 6, 13, and 15 in 2020 and population 15 in 2022, corrected survival of each field population was significantly greater than the LAB-S control within years (Tables 2–4). In the six fields with a long-term history of continuous maize and use of WCR-active Bt proteins (Fields 3, 7–10, 12), Bt pyramid corrected survival was consistently high but did not significantly change during the duration of this project (*F*_1,10_ = 0.06, *p* = 0.82; mean corrected survival: 2020: 0.58 ± 0.05; 2022: 0.56 ± 0.05). There was also significant variation in WCR susceptibility to Cry3Bb1 and Gpp34Ab1/Tpp35Ab1 among populations each year (Cry3Bb1: 2020: *F*_16,114.6_ = 59.34, *p* <0.0001; 2021: *F*_12,95.*2*_ = 106.63, *p* <0.0001; 2022: *F*_13,102.5_ = 71.21, *p* <0.0001; Gpp34Ab1/Tpp35Ab1: 2021: *F*_12,44.13_ = 28.73, *p* <0.0001; 2022: *F*_13,47.83_, *p <*0.0001; (Tables 2–4).

**Table 2:** Corrected survival of Nebraska western corn rootworm populations on Cry3Bb1 and Bt pyramid maize from 2020 larval bioassays.

Field No.	Cry3Bb1 Corrected Survival^c^^,^^d^	Bt Pyramid Corrected Survival^a^^,^^b^
**2**	0.866 ± 0.11abc	0.390 ± 0.11abcde
**3**	0.570 ± 0.09def	0.494 ± 0.09abc
**4**	0.776 ± 0.11bcd	0.421 ± 0.11abcde
**5**	1.063 ± 0.09a	0.367 ± 0.09bcde
**6**	0.467 ± 0.06fg	0.133 ± 0.06fg
**7**	0.805 ± 0.10bcd	0.597 ± 0.10ab
**8**	0.636 ± 0.08cdef	0.584 ± 0.08ab
**9**	0.724 ± 0.10bcde	0.658 ± 0.10a
**10**	0.816 ± 0.13abcd	0.571 ± 0.13abc
**12**	0.838 ± 0.08abc	0.427 ± 0.08abcd
**13**	0.096 ± 0.03h	0.000 ± 0.00h
**15**	0.290 ± 0.08g	0.158 ± 0.08efg
**18**	0.785 ± 0.07bcd	0.581 ± 0.07ab
**LAB-S**	0.036 ± 0.01h	0.021 ± 0.01gh

^a^Corrected survival = $1-\frac{survival\;on\;isoline-survival\;on\;Bt}{survvial\;on\;isoine}$.

^b^Bt pyramid = Cry3Bb1 + Gpp34Ab1/Tpp35Ab1.

^c^Gpp34Ab1/Tpp35Ab1 corrected survival for populations listed above published in [50] as 2020 populations 16–24, 26, 27, 29, 30, LAB-S, respectively.

^d^Corrected survival values followed by the same lowercase letter within a column are not significantly different (Tukey’s multiplicity adjustment, *P* >0.05).

**Table 3:** Corrected survival of Nebraska western corn rootworm populations on Cry3Bb1, Gpp34Ab1/Tpp35Ab1, and Bt pyramid maize from 2021 larval bioassays.

Field No.	Cry3Bb1 Corrected Survival^a^^,^^c^	Bt Pyramid Corrected Survival^b^	Gpp34Ab1/Tpp35Ab1 Corrected Survival
**2**	1.233 ± 0.18a	1.067 ± 0.18a	0.597 ± 0.08ab
**3**	1.116 ± 0.06a	0.837 ± 0.06ab	0.521 ± 0.07ab
**6**	0.795 ± 0.04c	0.274 ± 0.04e	0.197 ± 0.04c
**7**	0.827 ± 0.07bc	0.627 ± 0.07cd	0.433 ± 0.07b
**8**	0.713 ± 0.08c	0.913 ± 0.08a	0.658 ± 0.07a
**9**	0.807 ± 0.09bc	0.532 ± 0.09d	0.571 ± 0.07ab
**10**	1.048 ± 0.12ab	0.694 ± 0.12abcd	0.580 ± 0.09ab
**12**	0.877 ± 0.10abc	0.630 ± 0.10bcd	0.635 ± 0.14ab
**15**	0.211 ± 0.03d	0.127 ± 0.03f	0.181 ± 0.02c
**16**	0.191 ± 0.04d	0.107 ± 0.04f	0.173 ± 0.04c
**17**	0.835 ± 0.08bc	0.660 ± 0.08bcd	0.500 ± 0.09ab
**18**	1.052 ± 0.09ab	0.831 ± 0.09abc	0.582 ± 0.05ab
**LAB-S**	0.020 ± 0.01e	0.014 ± 0.01g	0.040 ± 0.01d

^a^Corrected survival = $1-\frac{survival\;on\;isoline-survival\;on\;Bt}{survvial\;on\;isoine}$.

^b^Bt pyramid = Cry3Bb1 + Gpp34Ab1/Tpp35Ab1.

^c^Corrected survival values followed by the same lowercase letter within a column are not significantly different (Tukey’s multiplicity adjustment, *p* >0.05).

**Table 4:** Corrected survival of Nebraska western corn rootworm populations on Cry3Bb1, Gpp34Ab1/Tpp35Ab1, and Bt pyramid maize from 2022 larval bioassays.

Field No.	Cry3Bb1 Corrected Survival^a^^,^^c^	Bt Pyramid Corrected Survival^b^	Gpp34Ab1/Tpp35Ab1 Corrected Survival
**2**	0.771 ± 0.09ab	0.313 ± 0.09c	0.228 ± 0.03d
**3**	0.750 ± 0.05ab	0.346 ± 0.05c	0.348 ± 0.05c
**4**	0.961 ± 0.09a	0.794 ± 0.09a	0.566 ± 0.05a
**6**	0.800 ± 0.14ab	0.675 ± 0.14ab	0.344 ± 0.05c
**7**	0.696 ± 0.08b	0.500 ± 0.08bc	0.419 ± 0.05abc
**8**	0.748 ± 0.07ab	0.505 ± 0.07bc	0.494 ± 0.08abc
**9**	0.724 ± 0.11ab	0.845 ± 0.11a	0.524 ± 0.06ab
**10**	0.866 ± 0.09ab	0.691 ± 0.09ab	0.595 ± 0.08a
**12**	0.660 ± 0.06b	0.564 ± 0.06b	0.391 ± 0.04bc
**15**	0.140 ± 0.04c	0.047 ± 0.04de	0.103 ± 0.02f
**16**	0.152 ± 0.02c	0.065 ± 0.02d	0.149 ± 0.03ef
**17**	0.720 ± 0.08b	0.550 ± 0.08b	0.214 ± 0.03de
**18**	0.800 ± 0.07ab	0.491 ± 0.07bc	0.500 ± 0.07abc
**LAB-S**	0.029 ± 0.01d	0.011 ± 0.01e	0.040 ± 0.01g

^a^Corrected survival = $1-\frac{survival\;on\;isoline-survival\;on\;Bt}{survvial\;on\;isoine}$.

^b^Bt pyramid = Cry3Bb1 + Gpp34Ab1/Tpp35Ab1.

^c^Corrected survival values followed by the same lowercase letter within a column are not significantly different (Tukey’s multiplicity adjustment, *p* >0.05).

### On-farm experiment: 2020–2022 composite analysis

**Fig 2:**
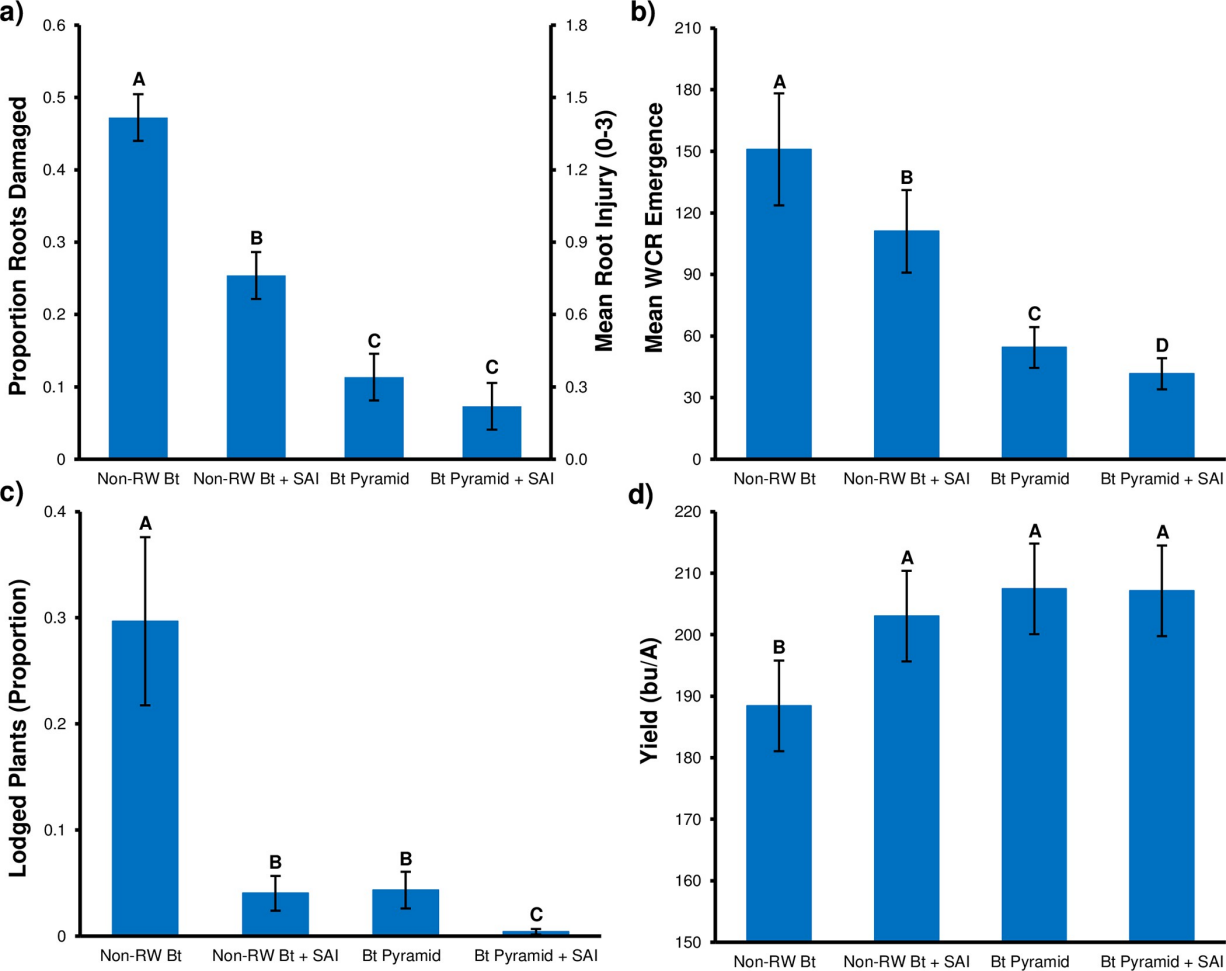
Results of generalized linear mixed model analysis to investigate effect of soil insecticide * hybrid treatments on corn rootworm and agronomic variables. (a) mean ± SE root injury rating: Proportion, average of 10 plants, normal distribution by Central Limit Theorem; (b) mean ± SE total adult emergence: Negative binomial with log link function; (c) Mean ± SE proportion lodged plants: Beta distribution; (d) Mean ± SE yield: Normal distribution. Number of sites = 34 except (d) N = 31 sites. Within a variable, LSMEANS with the same letter are not significantly different (SAS 9.4, GLMM; GLIMMIX procedure, LSMEANS with slice option; *p* >0.05).

**Root injury.** Mean root injury was significantly affected by the maize hybrid*soil insecticide interaction (*F*_1,9_ = 8.14; *p* = 0.019). Mean root injury was 1.42 in the non-RW Bt treatment across 34 fields and was significantly greater than mean root injury in the other treatments (Fig 2A). The addition of the SAI to non-RW maize significantly reduced root injury by 46%. The NIS ratings of the two Bt pyramid treatments were significantly lower (≤ 0.34 NIS, ≥ 76% reduction) than ratings in non-Bt maize strips. Mean NIS was not significantly different between Bt pyramid treatments (Fig 2A).

#### Adult emergence

Total WCR adult emergence was significantly affected by the main effects maize hybrid and soil insecticide (*F*_1,84_ = 1111.90, *p* <0.0001; and *F*_1,84_ = 9.87, *p* = 0.0023; respectively). Total adult production from each treatment followed the general pattern observed for root injury. Emergence from the non-RW Bt treatment was significantly greater than that recorded in the other treatments (Fig 2B). Within hybrids, the addition of the SAI significantly reduced mean emergence. The significant difference in emergence between Bt hybrid treatments differed from the NIS analysis which revealed a similar but nonsignificant trend. The Bt pyramid treatments reduced mean adult emergence by ≥ 63.9% (Fig 2B).

### Lodging

Mean plant lodging was significantly affected by the main effects maize hybrid and soil insecticide (*F*_1,84_ = 64.15, *p* <0.0001; *F*_1,84_ = 64.35, *p* <0.0001; respectively). The mean percent lodging recorded from the non-RW Bt treatment (mean = 29.7%) was significantly higher than lodging in the other treatments, which was minimal (≤ 4.3%) (Fig 2C). Within maize hybrids, the addition of the SAI significantly reduced percent lodging. The percent lodging was not significantly different between the non-RW Bt + SAI and Bt pyramid treatments (Fig 2C). The Bt pyramid + SAI reduced percent lodging to <0.4%.

#### Yield

Yield was significantly affected by the maize hybrid*soil insecticide interaction (*F*_1,84_ = 4.61, *p* = 0.034). Over multiple environments, mean grain yield was significantly reduced by 982–1278 kg/ha (14.6–19.0 bu/acre) in the non-RW-Bt treatment compared to the other treatments (Fig 2D). Mean yields of the non-RW Bt + SAI treatment and each Bt pyramid treatment were not significantly different (Fig 2D).

### On-farm experiment: Effect of WCR Bt resistance

#### Non-RW Bt: Bt pyramid analysis

*Mean NIS*. The non-RW Bt:Bt pyramid ratio of mean root injury ratings was significantly affected by corrected survival category (*F*_2,29_ = 14.39; *p* <0.0001). The mean NIS non-RW Bt:Bt pyramid ratio in the low corrected survival category was significantly greater than the ratios in the moderate and high corrected survival categories (Table 5). NIS ratios in moderate and high corrected survival categories were not significantly different (Table 5).

**Table 5:** Mean non-RW Bt: Bt hybrid ratio comparisons among corrected survival categories for the strip trial metrics NIS, total emergence, and yield.

Strip Trial Metric^a^	Corrected Survival Category^b^	N^c^	Mean non-RW Bt: Bt Pyramid (±SE)^c^
NIS	Low	8	21.46 (2.72) A
	Moderate	7	5.11 (2.90) B
	High	17	4.45 (1.86) B
Adult Emergence	Low	7	5.54 (1.10) A
	Moderate	7	5.30 (1.10) AB
	High	17	2.17 (0.70) B
Yield	Low	8	0.85 (0.05) A
	Moderate	6	0.84 (0.06) A
	High	15	0.95 (0.04) A

^a^ Adult emergence: Total emergence from 4 emergence cages per site; NIS: Mean 0–3 node injury score from 10 plants per site; grain yield at 15.5% moisture from 22.9 row m per treatment.

^b^ Corrected survival categories: Low: <0.3; moderate: ≥0.3–0.49; high: ≥0.5).

Within strip trial metric, LSMEANS with the same letter are not significantly different (One- way generalized linear model, PROC GLIMMIX in SAS; PDIFF option, *p* >0.05).

^c^N = number of sites.

*Adult emergence*. Corrected survival categories significantly affected the non-RW Bt:Bt pyramid adult emergence ratio (*F*_2,28_ = 4.81, *p* = 0.016). The mean total adult emergence non-RW Bt:Bt pyramid ratio in the low corrected survival category was significantly larger than the ratio in the high corrected survival category (Table 5). The emergence non-RW Bt:Bt pyramid ratio in the moderate corrected survival category was not significantly different than ratios in the other two categories (Table 5).

*Yield*. The mean non-RW Bt:Bt pyramid yield ratio was not significantly affected by corrected survival categories (*F*_2,26_ = 1.89, *p* = 0.172). Therefore, the mean yield non-RW Bt:Bt pyramid ratio was not significantly different among corrected survival categories (Table 5).

### Non-RW Bt x Bt pyramid linear regressions

**Fig 3:**
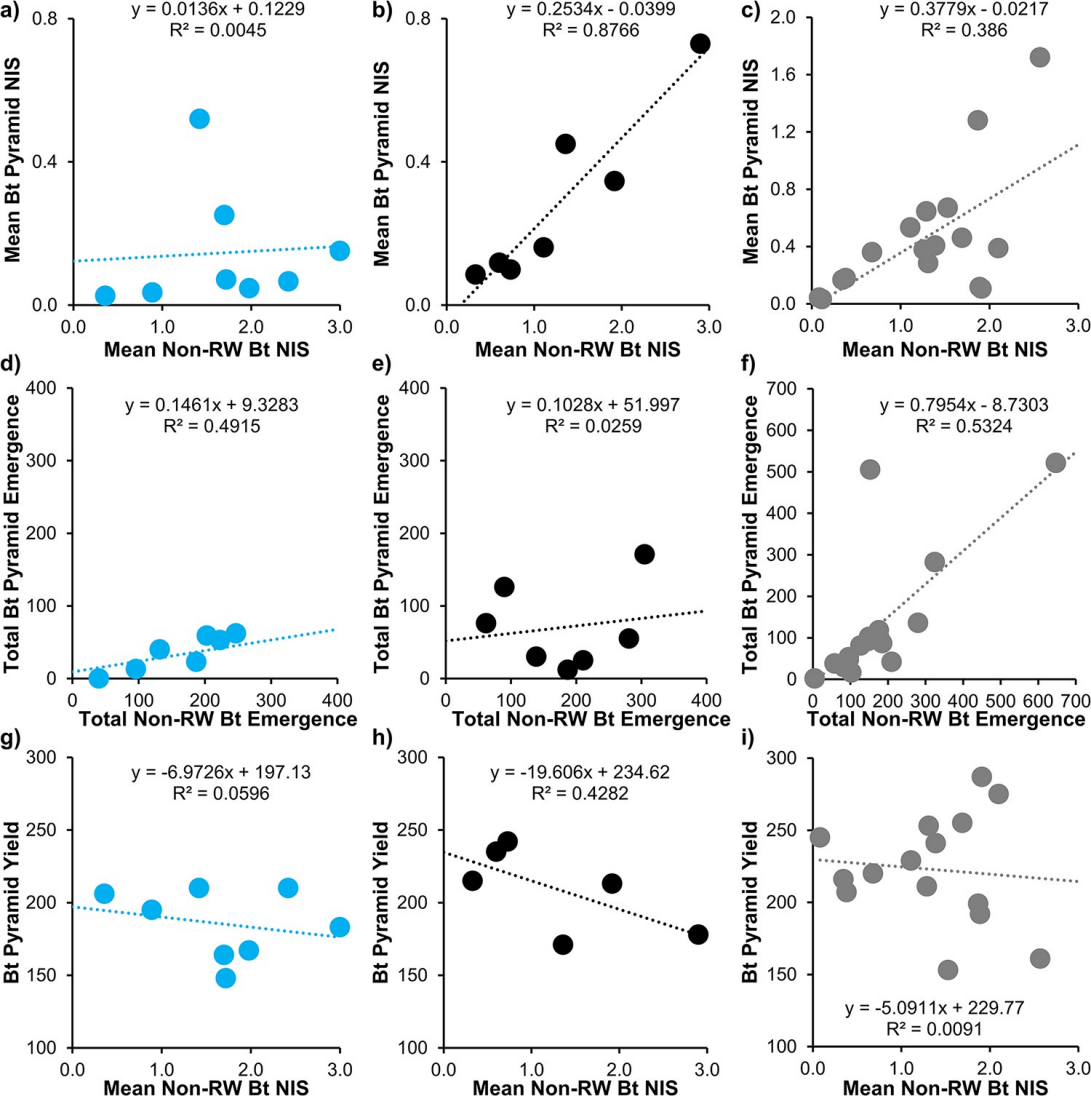
Linear regression of non-RW Bt and Bt pyramid strip trial variables for each Bt hybrid bioassay corrected survival category (<0.3 blue dots [a, d, g], 0.3–0.49 black dots [b, e, h], >0.5 gray dots [c, f, i]). (a-c): Mean NIS; (d-f) Total adult emergence; (g-i) Mean non-RW Bt NIS x Bt pyramid yield.

*Mean NIS*. The regression of non-RW Bt NIS x mean Bt pyramid NIS at the low corrected survival category was not significant (*F*_1,6_ = 0.03, *p* = 0.875). Mean NIS did not significantly change as non-RW Bt root injury increased (Fig 3A). *R*^*2*^ (0.005) and Pearson’s correlation coefficient (*r* = 0.067) were very low. In the moderate and high corrected survival categories, regressions were highly significant (moderate: *F*_1,5_ = 35.51, *p* = 0.0019; high: *F* = 9.43, *p* = 0.0078). In both cases, mean Bt pyramid NIS increased as mean NIS of non-RW Bt increased (Fig 3B and 3C). *R*^*2*^ and *r* were intermediate to high (moderate: *R*^*2*^ = 0.88, *r* = 0.94; high: *R*^*2*^ = 0.39, *r* = 0.62).

*Total adult emergence*. Linear regressions of adult emergence from non-RW Bt x Bt pyramid in the low and moderate corrected survival categories were not significant (low: *F*_1,6_ = 5.80, *p* = 0.053; moderate: *F*_1,5_ = 0.13, *p* = 0.730). *R*^*2*^ values were low-intermediate (low: *R*^*2*^ = 0.49; moderate: *R*^*2*^ = 0.026) with variable strength of association between treatments (low: *r* = 0.70; moderate: *r* = 0.16). In both low and moderate corrected survival categories, total Bt pyramid emergence remained at a relatively consistent low level as non-RW Bt emergence increased (Fig 3D and 3E). Linear regression was highly significant in the high corrected survival category (*F*_1,15_ = 17.08, *p* = 0.0009, *R*^*2*^ = 0.53) and strength of association between treatments was high (*r* = 0.73). Total emergence from the Bt pyramid increased as emergence from non-RW Bt increased (Fig 3F). If the lone outlier was removed, *R*^*2*^ and *r* increased to 0.92 and 0.96, respectively.

*Grain yield*. Regressions of non-RW Bt NIS x Bt pyramid yield in all corrected survival categories were not statistically significant (low: *F*_1,6_ = 0.38, *p* = 0.56; moderate: *F*_1,4_ = 2.99, *p* = 0.16; high: *F*_1,13_ = 0.12, *p* = 0.74; Fig 3G–3I). *R*^*2*^ values were low-intermediate (low: *R*^*2*^ = 0.06, moderate: *R*^*2*^ = 0.43, high R^2^: 0.01) with negative low-intermediate strength of association between treatments (low: *r* = -0.24; moderate: *r* = -0.65, high: *r* = -0.10).

## Discussion

Data from this study provide examples of WCR practical resistance (defined by Tabashnik et al. [63]) to the Bt pyramid and the WCR density x resistance level interactions that impact Bt pyramid root injury and adult emergence. WCR practical resistance to the Bt pyramid has also been documented in Iowa [39] and North Dakota [49]. Most field populations in this study exhibited incomplete resistance (i.e., significantly higher survival on Bt maize than susceptible laboratory control populations and significantly greater survival and/or development metrics on non-Bt than Bt maize [64]) to the Bt pyramid (Tables 2–4). WCR resistance to the Bt pyramid has been present in the northeast Nebraska region since at least 2017 [47]. A complex interaction of population and landscape factors determines WCR population susceptibility to Bt proteins (reviewed by Gassmann [52]). In the study area, the mosaic of agricultural practices (i.e., clusters of hectares in continuous maize) and variability in WCR management tactics were key factors which can impact WCR densities, movement of resistant alleles, and level of Bt selection pressure at the field and farm levels [46,65,66]. The documented variability in WCR susceptibility to single-protein Cry3Bb1 and Gpp34Ab1/Tpp35Ab1 among populations in the region ([47,50]; this study: Tables 2–4) leads to differences in redundant killing potential of the Bt pyramid among populations, which can also influence the rate of resistance evolution to the Bt pyramid [52,67,68].

Root injury is a key variable that was measured since it is associated with larval pressure and related to subsequent adult emergence and the agronomic parameters lodging and yield. The landscape level analysis clearly showed the value of the Bt pyramid treatments to significantly reduce root injury compared to non-RW Bt treatments. This was especially apparent when little to no WCR Bt resistance was present (Bt pyramid corrected survival <0.3), which was independent of WCR density. This complements results from a recent study that reported mean Bt pyramid NIS <0.04 when rootworm pressure was extreme (non-RW Bt NIS: >2.5) and corrected survival was 0.047 [69]. In contrast, when moderate to high levels of resistance were present, results provided evidence that a WCR density level x resistance level interaction occurs, facilitating an increasing reduction in the level of control from the Bt pyramid as density (larval pressure) increases (Fig 3B and 3C).

Similar trends were observed between NIS and adult emergence in several analyses. The Bt pyramid greatly reduced mean adult emergence compared to non-RW Bt in the landscape level analysis. When WCR resistance was low, adult emergence from the Bt pyramid remained consistently low as emergence increased in the non-RW Bt treatment. In contrast, when WCR resistance level was high, emergence patterns tracked more closely between hybrids as non-RW Bt emergence increased (Fig 3D–3F). These trends also reveal the importance of the WCR density x resistance interaction that impacts adult emergence. Variability in results occurred in some fields at high densities where density-dependent mortality can negatively impact total emergence [70]. For example, Field 10 (2022) total emergence from four emergence cages was 153, 606, 505 and 731 in non-RW Bt, non-RW Bt + SAI, Bt pyramid, and Bt pyramid + SAI treatments, respectively (S3 Table). The SAI and Bt pyramid treatments reduced the larval population and density-dependent mortality of remaining individuals, which led to higher survival to the adult stage (e.g., outlier Fig 3F). This phenomenon has also been observed when conventional soil insecticides are applied when WCR densities are high [71].

Yield trends were different than previously described for NIS and adult emergence. In the landscape analysis, yield was only significantly reduced in the non-RW Bt treatment. In addition, the density level x resistance level interaction was not observed in analyses of yield that included different levels of resistance. Results indicate that Bt pyramid resistance levels present in many of the fields included in this study were not high enough to create a negative Bt pyramid yield response under moderate-high rootworm pressure. Results were conservative as lodging was extensive in some non-RW Bt plots (S1–S3 Tables), which may have further reduced non-RW Bt yield if plots had been machine-harvested.

Reports in the literature on WCR efficacy (NIS) of the Bt pyramid or associated adult emergence have primarily been limited to populations that were not resistant to the Bt pyramid. Results of four large multi-year studies conducted across the midwestern U.S. clearly showed the Bt pyramid significantly reduced mean NIS to very low levels (often NIS ≤ 0.1) compared to a non-RW Bt control, which included some sites with suspected resistance to Cry3Bb1 [33,53,72,73]. Mean Bt pyramid NIS was consistently low in the Head et al. [33] and Johnson et al. [73] studies at both low and high WCR densities. This trend was similar to the NIS trend observed in this study from the Bt pyramid low corrected survival category (Fig 3A). These studies were primarily conducted prior to documented WCR evolution of resistance to Gpp34Ab1/Tpp35Ab1 [38,39,50] and the Bt pyramid in the field [39,47,49]. Head et al. [53] reported low mean NIS in the Gpp34Ab1/Tpp35Ab1 treatment relative to the non-RW Bt control at all high WCR pressure sites except for one population from Cuming Co., NE where mean Gpp34Ab1/Tpp35Ab1, Cry3Bb1, and Bt pyramid NIS were not significantly different than the mean non-RW Bt NIS. This suggested possible WCR resistance to the Bt pyramid was evolving in Nebraska. In contrast, most populations in this study exhibited some level of resistance to Cry3Bb1, Gpp34Ab1/Tpp35Ab1, and the Bt pyramid (Tables 2–4).

Multiple studies have documented that the Bt pyramid often reduces WCR survival to the adult stage to < 1–2% when WCR populations are susceptible to Bt proteins [33,34,53,74,75]. In this study, after additional years of selection with both Cry3 or Gpp34Ab1/Tpp35Ab1 and Cry3 + Gpp34Ab1/Tpp35Ab1 pyramids, mean survival to adulthood in the low, moderate, and high Bt pyramid corrected survival categories in relation to the non-RW Bt treatment was 19.5%, 38.8%, and 74.6%, respectively, providing additional evidence that WCR resistance to Bt traits has generally increased in the landscape over the last decade.

Rootworm management options are currently limited in the continuous maize production system, leading to trade-offs between IPM and IRM objectives. Mean non-RW Bt NIS was > 1.0 in 70% of commercial maize fields, which indicates annual WCR control measures were warranted in most fields. Yield response to WCR injury can be quite variable because it is driven by the larval density, hybrid genetics, and environmental interaction [19]. Yield reduction per node of root injury was 9.7–12% across multiple hybrids in an irrigated Nebraska study [19] while meta-analyses of non-irrigated data suggest the potential for a 15–18% yield reduction per node of injury [21,76]. The mean Bt pyramid NIS remained below 0.75 in all but two fields with high mean NIS (Field 12, 2021; Field 10, 2022), which helps explain the lack of a general Bt pyramid yield response in any corrected survival category. Gassmann et al. [39] also reported two commercial fields with extreme pressure and very high Bt pyramid mean NIS in Iowa after many years of Bt pyramid use in continuous maize.

Most farmers in the study area annually plant a Cry3 + Gpp34Ab1/Tpp35Ab1 pyramid and a subset routinely apply a planting-time soil insecticide with a Bt pyramid, especially if they had experienced WCR resistance to single Cry3 proteins with associated crop injury and lodging (Meinke personal observation). This results in continuous Bt protein selection pressure placed on the resident WCR population, which conflicts with IRM objectives [77]. Results from this study indicate that the addition of a soil insecticide will not significantly improve Bt pyramid root protection or yield. Unless soil insect pests other than WCR are the primary concern, the economic investment in a soil insecticide for extra root and yield protection is questionable. Similar results with Bt pyramid + a soil insecticide were obtained in previous studies for variables NIS [73,78] and yield when WCR Bt pyramid resistance was absent and NIS was ≤ 2.0 [73]. In contrast, the non-RW Bt + SAI and Bt pyramid + SAI both significantly reduced lodging and adult emergence in this study. Sutter et al. [79] also reported a conventional hybrid + soil insecticide significantly reduced lodging. An effective soil insecticide would greatly reduce the lodging risk associated with refuge plants or Bt pyramid plants in a high resistance/high density environment. In this study, reduction in mean WCR adult emergence was 23.6% when comparing Bt pyramid + SAI to the Bt pyramid. This result could potentially benefit IRM, but it is unclear if this difference would significantly reduce egg production or the number of WCR resistant individuals produced.

In the continuous maize production system, periodic rotation from a Bt pyramid to a non-RW-Bt hybrid + SAI would also present some tradeoffs. In this study, yield and potential lodging risk were not significantly different between non-RW Bt + SAI and Bt pyramid treatments so the economic risk could be minimal. WCR survival in a field will usually increase with rotation to a non-RW hybrid, which could have positive or negative effects on IPM and IRM. This agronomic approach would probably be best from an IRM standpoint to slow the evolution of resistance when a new technology is introduced or WCR resistance is relatively low. Rotating from Bt pyramid maize to non-RW Bt maize + soil insecticide when WCR resistance is low would break the cycle of continuous selection with Bt traits and potentially increase the number of adults contributing susceptible alleles to the next generation. In contrast, when the frequency of Bt-resistant WCR individuals is high, this approach would only increase the number of resistant individuals in a population and potential R x R mating since WCR Bt resistance can be maintained in a population for some time after removal of selection [55,80,81]. This approach may increase selection pressure if a Bt pyramid was planted the following season. Another potential negative would be silk-clipping by adults when densities are high, which could interfere with pollination under certain conditions [82].

The effect of WCR pressure level x level of WCR Bt resistance interaction on efficacy of the Bt pyramid and subsequent adult emergence is a key result of this study, which can also lead to IPM and IRM tradeoffs. In the moderate corrected survival category, when mean NIS level was extreme (near 3.0) the mean Bt pyramid NIS was also elevated but still <0.75 (Fig 2B), which from a rootworm management standpoint may not significantly reduce yield in irrigated maize. However, the density x resistance interaction increased the mean NIS level over the greater than expected injury threshold set by the U.S. Environmental Protection Agency (NIS ≥ 0.5 for pyramids [36,83]), which triggers industry investigation if farmer reported [77]. Across Bt pyramid bioassay corrected survival levels recorded in this study, the mean Bt pyramid NIS exceeded the EPA trigger of 0.5 in six fields. The regressions of WCR populations in medium-high corrected survival categories where the greater than expected injury trigger was exceeded generally occurred when the non-RW Bt NIS was >1.4 (Fig 2C). This was also observed in a recent study where mean non-RW Bt NIS was ca. 1.4 but high WCR Bt pyramid resistance (bioassay corrected survival = 0.84) increased the mean Bt pyramid NIS to 0.67 [69]. IPM and IRM conflict can occur when Bt pyramid NIS is >0.5 but <0.75 in this system. Larval injury exceeds the IRM trigger but unless the grower is aware of increasing injury or lodging and does not see a significant yield reduction there is little incentive to move away from continuous planting of Bt pyramid maize.

Field 10 is a good case history that demonstrates the impact of WCR resistance at different densities when WCR resistance is high and IPM and IRM are in conflict. In 2020, low pressure in non-RW Bt and the Bt pyramid (non-RW Bt mean NIS: 0.68 ± 0.14; Bt pyramid mean NIS: 0.36 ± 0.06; Bt pyramid bioassay corrected survival: 0.57) masked the resistance level present and provided acceptable injury management. In 2021, mean NIS in the non-RW Bt treatment increased (mean NIS: 1.53 ± 0.12) and Bt pyramid mean NIS increased above 0.5 but still provided acceptable injury management while maintaining the high Bt pyramid resistance level (mean NIS: 0.67 ± 0.13, Bt pyramid bioassay corrected survival: 0.69). In 2022, mean NIS increased to 2.57 ± 0.21 and 1.72 ± 0.22 in the non-RW Bt and Bt pyramid treatments, respectively, while maintaining resistance level (Bt pyramid corrected survival: 0.69), which greatly exceeded the IRM threshold and did not provide adequate injury management. This example also demonstrates how quickly WCR densities can increase in continuous maize when a high level of Bt pyramid resistance is present. Fitness costs associated with WCR Bt resistance are often minimal and inheritance of resistance is often non-recessive [reviewed in 52,84] which may have facilitated the rapid density increase observed.

This study is the first to document the importance of the WCR larval pressure x WCR resistance interaction on Bt pyramid efficacy, adult emergence, and yield, which revealed tradeoffs between IPM and IRM in the continuous maize production system. The significant linear increases in mean Bt pyramid NIS and emergence as mean non-RW Bt NIS increased were key results when high Bt pyramid resistance levels were present. In areas with clusters of continuous maize and a high frequency of WCR individuals resistant to Bt pyramids, it is too late to remediate resistance from the landscape, but farmers can manage WCR densities. Under this scenario, a number of tactics can be used to alter the impact of the WCR density x resistance interaction. Well-timed foliar insecticide applications targeting adult WCR females [82,85] or potential use of nematodes [86,87] are options to complement pyramids in continuous maize. Another consideration in the continuous maize system is the recently commercialized triple pyramid Cry3Bb1 + Gpp34Ab1/Tpp35Ab1 + DvSnf7 dsRNA [53]. This technology reduced WCR survival to the adult stage by 97–99% in a recent field study conducted where WCR exhibited high levels of resistance to Cry3Bb1 and Gpp34Ab1/Tpp35Ab1 [88]. Use of this technology to greatly lower density followed by rotation to a Bt pyramid would help slow WCR resistance evolution to the RNAi trait and reduce potential injury to the Bt pyramid. The downside would be the continual selection for resistance to Cry3Bb1 and Gpp34Ab1/Tpp35Ab1. Periodic rotation to a non-host crop (e.g., soybeans) is the preferred way to reduce WCR densities and alter the impact of the density x resistance interaction. Crop rotation will remove resident WCR from a field for one year but reinfestation with resistant adult WCR from adjacent fields can occur in first-year maize [46; this study: Fields 17,18]. Reducing long-term use of continuous maize from > 4–10+ years to 2–3 years maize: 1 year non-host crop (e.g. soybeans) would enable non-RW Bt to be planted after the rotational crop, thus removing pyramid selection pressure for 2 consecutive years before returning to a transgenic pyramid in second-year maize. More frequent rotation at the individual farm level coupled with pyramid use could also potentially reduce WCR density in the landscape over time. In summary, use of pyramid technologies within an IPM framework may enable farmers to slow the evolution of WCR resistance to new traits [47,77,89], manage the impact of Bt resistance by reducing WCR densities, and bridge use of current pyramids with new technologies that will be introduced over the next decade.

## Supporting information

S1 Table Total emergence, mean root injury, proportion lodged plants, and yield from each strip trial treatment per site, 2020. (DOCX)

S2 Table Total emergence, mean root injury, proportion lodged plants, and yield from each strip trial treatment per site, 2021. (DOCX)

S3 Table Total emergence, mean root injury, proportion lodged plants, and yield from each strip trial treatment per site, 2022. (DOCX)

S4 Table Mean proportional survival (± SE) of susceptible lab control colonies in 2020 bioassays. (A) Cry3Bb1, (B) Cry3Bb1 + Gpp34Ab1/Tpp35Ab1 pyramid. Within hybrids, no significant differences in mean survival among colonies were documented (GLMM, binomial distribution; *P*>0.05).
(DOCX)

S5 Table Mean proportional survival (± SE) of susceptible lab control colonies in 2021 bioassays. (A) Cry3Bb1, (B) Gpp34Ab1/Tpp35Ab1, (C) Cry3Bb1 + Gpp34Ab1/Tpp35Ab1 pyramid. Within hybrids, no significant differences in mean survival among colonies were documented (GLMM, binomial distribution; *P*>0.05).
(DOCX)

S6 Table Mean proportional survival (± SE) of susceptible lab control colonies in 2022 bioassays. (A) Cry3Bb1, (B) Gpp34Ab1/Tpp35Ab1, (C) Cry3Bb1 + Gpp34Ab1/Tpp35Ab1 pyramid. Within hybrids, no significant differences in mean survival among colonies were documented (GLMM, binomial distribution; *P*>0.05).
(DOCX)
