# Supplementary material for: Resistance management and integrated pest management insights from deployment of a Cry3Bb1+ Gpp34Ab1/Tpp35Ab1 pyramid in a resistant western corn rootworm landscape
Source: PLoS One. 2024 Mar 8;19(3):e0299483. doi: 10.1371/journal.pone.0299483 (PMC10923451; doi:10.1371/journal.pone.0299483)
Supplement: S1 Table — (DOCX) [file pone.0299483.s001.docx]

S1.1 Table. Total emergence, mean root injury, proportion lodged plants, and yield from each strip trial treatment, 2020.

|  |  | **2020 Strip Trial Variables** | | | |
| --- | --- | --- | --- | --- | --- |
| Field | Treatment | Total Emergence | Root Injury (0-3 NIS) | Lodged Plants | Yield (bu/A) |
| 2 | Non-RW Bt | 281 | 1.92 ± 0.19 | 0.352 | 146 |
|  | Non-RW Bt + SAI | 141 | 0.91 ± 0.14 | 0.000 | 212 |
|  | Bt Pyramid | 56 | 0.35 ± 0.08 | 0.000 | 213 |
|  | Bt Pyramid + SAI | 42 | 0.18 ± 0.04 | 0.000 | 220 |
| 3 | Non-RW Bt | 130 | 0.73 ± 0.23 | 0.017 | 189 |
|  | Non-RW Bt + SAI | 67 | 0.17 ± 0.06 | 0.017 | 196 |
|  | Bt Pyramid | 30 | 0.10 ± 0.03 | 0.000 | 242 |
|  | Bt Pyramid + SAI | 19 | 0.04 ± 0.01 | 0.007 | 194 |
| 5 | Non-RW Bt | 188 | 1.11 ± 0.17 | 0.000 | 63 |
|  | Non-RW Bt + SAI | 34 | 0.34 ± 0.07 | 0.000 | 54 |
|  | Bt Pyramid | 12 | 0.16 ± 0.04 | 0.000 | 91 |
|  | Bt Pyramid + SAI | 26 | 0.07 ± 0.01 | 0.000 | 89 |
| 6 | Non-RW Bt | 401 | 1.42 ± 0.17 | 0.712 | 164 |
|  | Non-RW Bt + SAI | 147 | 0.95 ± 0.11 | 0.098 | 208 |
|  | Bt Pyramid | 48 | 0.52 ± 0.11 | 0.121 | 210 |
|  | Bt Pyramid + SAI | 45 | 0.21 ± 0.05 | 0.000 | 219 |
| 7 | Non-RW Bt | 176 | 1.29 ± 0.08 | 0.075 | 201 |
|  | Non-RW Bt + SAI | 261 | 0.66 ± 0.09 | 0.008 | 157 |
|  | Bt Pyramid | 118 | 0.61 ± 0.13 | 0.000 | 211 |
|  | Bt Pyramid + SAI | 61 | 0.10 ± 0.01 | 0.000 | 164 |
| 8 | Non-RW Bt | 185 | 0.38 ± 0.11 | 0.000 | 191 |
|  | Non-RW Bt + SAI | 175 | 0.32 ± 0.07 | 0.000 | 212 |
|  | Bt Pyramid | 87 | 0.18 ± 0.05 | 0.000 | 207 |
|  | Bt Pyramid + SAI | 52 | 0.07 ± 0.01 | 0.000 | 192 |
| 10 | Non-RW Bt | 177 | 0.68 ± 0.14 | 0.214 | 231 |
|  | Non-RW Bt + SAI | 113 | 0.29 ± 0.10 | 0.007 | 250 |
|  | Bt Pyramid | 101 | 0.36 ± 0.06 | 0.357 | 220 |
|  | Bt Pyramid + SAI | 95 | 0.22 ± 0.09 | 0.000 | 223 |
| 12 | Non-RW Bt | 305 | 1.36 ± 0.17 | 0.137 | 152 |
|  | Non-RW Bt + SAI | 363 | 1.13 ± 0.09 | 0.133 | 147 |
|  | Bt Pyramid | 171 | 0.45 ± 0.12 | 0.121 | 171 |
|  | Bt Pyramid + SAI | 182 | 0.31 ± 0.05 | 0.000 | 176 |
| 13 | Non-RW Bt | 40 | 0.36 ± 0.19 | 0.000 | 202 |
|  | Non-RW Bt + SAI | 73 | 0.14 ± 0.04 | 0.000 | 209 |
|  | Bt Pyramid | 0 | 0.03 ± 0.01 | 0.000 | 163 |
|  | Bt Pyramid + SAI | 3 | 0.03 ± 0.01 | 0.000 | 206 |
| 15 | Non-RW Bt | 247 | 1.72 ± 0.17 | 0.595 | 134 |
|  | Non-RW Bt + SAI | 99 | 0.15 ± 0.04 | 0.000 | 167 |
|  | Bt Pyramid | 62 | 0.07 ± 0.01 | 0.000 | 148 |
|  | Bt Pyramid + SAI | 59 | 0.05 ± 0.01 | 0.000 | 156 |
| 16 | Non-RW Bt | 109 | 0.71 ± 0.09 | 0.000 | 196 |
|  | Non-RW Bt + SAI | 128 | 0.29 ± 0.09 | 0.000 | 193 |
|  | Bt Pyramid | 19 | 0.04 ± 0.01 | 0.000 | 212 |
|  | Bt Pyramid + SAI | 14 | 0.04 ± 0.01 | 0.000 | 203 |

Non-RW Bt: no corn rootworm traits; Bt pyramid: Cry3Bb1 + Gpp34Ab1/Tpp35Ab1; SAI: soil-applied insecticide; Total emergence from 4 single-plant emergence cages per treatment/site; NIS: 0-3 node injury scale, N=10 roots per treatment /site; Lodged plants: Mean proportion of plants leaning ≥ 45^o^ from stalk; Yield from 22.9 row-m per treatment.
