# Supplementary material for: Resistance management and integrated pest management insights from deployment of a Cry3Bb1+ Gpp34Ab1/Tpp35Ab1 pyramid in a resistant western corn rootworm landscape
Source: PLoS One. 2024 Mar 8;19(3):e0299483. doi: 10.1371/journal.pone.0299483 (PMC10923451; doi:10.1371/journal.pone.0299483)
Supplement: S2 Table — (DOCX) [file pone.0299483.s002.docx]

S1.2 Table. Total emergence, mean root injury, proportion lodged plants, and yield from each strip trial treatment, 2021.

|  |  | **2021 Strip Trial Variables** | | | |
| --- | --- | --- | --- | --- | --- |
| Field | Treatment | Total Emergence | Root Injury (0-3 NIS) | Lodged Plants | Yield (bu/A) |
| 2 | Non-RW Bt | 96 | 1.69 ± 0.25 | 0.119 | 288 |
|  | Non-RW Bt + SAI | 74 | 0.63 ± 0.08 | 0.058 | 246 |
|  | Bt Pyramid | 53 | 0.46 ± 0.09 | 0.029 | 255 |
|  | Bt Pyramid + SAI | 25 | 0.22 ± 0.05 | 0.014 | 255 |
| 3 | Non-RW Bt | 5 | 0.08 ± 0.01 | 0.000 | 257 |
|  | Non-RW Bt + SAI | 2 | 0.07 ± 0.01 | 0.000 | 224 |
|  | Bt Pyramid | 2 | 0.04 ± 0.01 | 0.000 | 245 |
|  | Bt Pyramid + SAI | 0 | 0.05 ± 0.01 | 0.000 | 221 |
| 6 | Non-RW Bt | 203 | 1.70 ± 0.20 | 0.248 | 162 |
|  | Non-RW Bt + SAI | 256 | 1.34 ± 0.20 | 0.054 | 130 |
|  | Bt Pyramid | 59 | 0.25 ± 0.06 | 0.000 | 164 |
|  | Bt Pyramid + SAI | 16 | 0.20 ± 0.07 | 0.000 | 144 |
| 7 | Non-RW Bt | 58 | 1.31 ± 0.14 | 0.520 | 203 |
|  | Non-RW Bt + SAI | 63 | 0.44 ± 0.09 | 0.000 | 248 |
|  | Bt Pyramid | 38 | 0.28 ± 0.14 | 0.008 | 253 |
|  | Bt Pyramid + SAI | 18 | 0.08 ± 0.02 | 0.000 | 218 |
| 8 | Non-RW Bt | 127 | 1.11 ± 0.16 | 0.000 | 168 |
|  | Non-RW Bt + SAI | 99 | 0.81 ± 0.22 | 0.000 | 244 |
|  | Bt Pyramid | 81 | 0.53 ± 0.13 | 0.000 | 229 |
|  | Bt Pyramid + SAI | 23 | 0.16 ± 0.07 | 0.000 | 204 |
| 9 | Non-RW Bt | 94 | 2.10 ± 0.10 | 0.376 | 240 |
|  | Non-RW Bt + SAI | 53 | 1.16 ± 0.16 | 0.086 | 238 |
|  | Bt Pyramid | 47 | 0.39 ± 0.08 | 0.008 | 275 |
|  | Bt Pyramid + SAI | 47 | 0.22 ± 0.07 | 0.000 | 276 |
| 10 | Non-RW Bt | 325 | 1.53 ± 0.12 | 0.475 | 175 |
|  | Non-RW Bt + SAI | 287 | 0.84 ± 0.16 | 0.017 | 184 |
|  | Bt Pyramid | 282 | 0.67 ± 0.13 | 0.252 | 153 |
|  | Bt Pyramid + SAI | 210 | 0.60 ± 0.12 | 0.056 | 187 |
| 12 | Non-RW Bt | 280 | 1.87 ± 0.18 | 0.733 | 227 |
|  | Non-RW Bt + SAI | 186 | 1.46 ± 0.09 | 0.669 | 196 |
|  | Bt Pyramid | 136 | 1.29 ± 0.16 | 0.387 | 199 |
|  | Bt Pyramid + SAI | 104 | 0.66 ± 0.13 | 0.038 | 204 |
| 15 | Non-RW Bt | 223 | 3.00 ± 0.00 | 1.000 | 105 |
|  | Non-RW Bt + SAI | 108 | 1.50 ± 0.07 | 0.023 | 181 |
|  | Bt Pyramid | 53 | 0.15 ± 0.05 | 0.295 | 183 |
|  | Bt Pyramid + SAI | 12 | 0.14 ± 0.04 | 0.000 | 167 |
| 16 | Non-RW Bt | 96 | 2.42 ± 0.16 | 0.923 | 162 |
|  | Non-RW Bt + SAI | 26 | 1.22 ± 0.12 | 0.031 | 199 |
|  | Bt Pyramid | 13 | 0.07 ± 0.01 | 0.016 | 210 |
|  | Bt Pyramid + SAI | 7 | 0.06 ± 0.02 | 0.000 | 209 |
| 17 | Non-RW Bt | 150 | 1.89 ± 0.06 | 1.000 | 179 |
|  | Non-RW Bt + SAI | 176 | 0.84 ± 0.11 | 0.623 | 195 |
|  | Bt Pyramid | 102 | 0.12 ± 0.02 | 0.293 | 192 |
|  | Bt Pyramid + SAI | 59 | 0.14 ± 0.03 | 0.113 | 213 |

Non-RW Bt: no corn rootworm traits; Bt pyramid: Cry3Bb1 + Gpp34Ab1/Tpp35Ab1; SAI: soil-applied insecticide; Total emergence from 4 single-plant emergence cages per treatment/site; NIS: 0-3 node injury scale, N=10 roots per treatment /site; Lodged plants: Mean proportion of plants leaning ≥ 45^o^ from stalk; Yield from 22.9 row-m per treatment.
