# Supplementary material for: Resistance management and integrated pest management insights from deployment of a Cry3Bb1+ Gpp34Ab1/Tpp35Ab1 pyramid in a resistant western corn rootworm landscape
Source: PLoS One. 2024 Mar 8;19(3):e0299483. doi: 10.1371/journal.pone.0299483 (PMC10923451; doi:10.1371/journal.pone.0299483)
Supplement: S3 Table — (DOCX) [file pone.0299483.s003.docx]

S1.3 Table. Total emergence, mean root injury, proportion lodged plants, and yield from each strip trial treatment. 2022.

|  |  | **2022 Strip Trial Variables** | | | |
| --- | --- | --- | --- | --- | --- |
| Field | Treatment | Total Emergence | Root Injury (0-3 NIS) | Lodged Plants | Yield (bu/A) |
| 2 | Non-RW Bt | 211 | 0.33 ± 0.09 | 0.000 | 200 |
|  | Non-RW Bt + SAI | 115 | 0.50 ± 0.12 | 0.000 | 200 |
|  | Bt Pyramid | 25 | 0.09 ± 0.02 | 0.000 | 215 |
|  | Bt Pyramid + SAI | 71 | 0.07 ± 0.02 | 0.000 | 197 |
| 3 | Non-RW Bt | 62 | 0.60 ± 0.14 | 0.000 | 220 |
|  | Non-RW Bt + SAI | 31 | 0.31 ± 0.09 | 0.000 | 221 |
|  | Bt Pyramid | 76 | 0.12 ± 0.02 | 0.000 | 235 |
|  | Bt Pyramid + SAI | 66 | 0.09 ± 0.02 | 0.000 | 221 |
| 4 | Non-RW Bt | 83 | 0.11 ± 0.07 | 0.000 | 70 |
|  | Non-RW Bt + SAI | 147 | 0.05 ± 0.01 | 0.000 | 99 |
|  | Bt Pyramid | 29 | 0.03 ± 0.01 | 0.000 | 108 |
|  | Bt Pyramid + SAI | 60 | 0.06 ± 0.02 | 0.000 | 97 |
| 7 | Non-RW Bt | 210 | 1.91 ± 0.16 | 0.651 | 258 |
|  | Non-RW Bt + SAI | 132 | 0.80 ± 0.20 | 0.174 | 259 |
|  | Bt Pyramid | 42 | 0.11 ± 0.03 | 0.142 | 287 |
|  | Bt Pyramid + SAI | 63 | 0.20 ± 0.04 | 0.000 | 297 |
| 8 | Non-RW Bt | 147 | 0.35 ± 0.14 | 0.256 | 217 |
|  | Non-RW Bt + SAI | 53 | 0.06 ± 0.01 | 0.000 | 201 |
|  | Bt Pyramid | 92 | 0.17 ± 0.05 | 0.153 | 216 |
|  | Bt Pyramid + SAI | 58 | 0.06 ± 0.01 | 0.000 | 233 |
| 9 | Non-RW Bt | 103 | 1.39 ± 0.11 | 0.353 | 217 |
|  | Non-RW Bt + SAI | 59 | 0.58 ± 0.07 | 0.000 | 225 |
|  | Bt Pyramid | 16 | 0.41 ± 0.12 | 0.233 | 241 |
|  | Bt Pyramid + SAI | 8 | 0.08 ± 0.02 | 0.000 | 231 |
| 10 | Non-RW Bt | 153 | 2.57 ± 0.21 | 0.781 | 107 |
|  | Non-RW Bt + SAI | 606 | 2.37 ± 0.23 | 0.409 | 101 |
|  | Bt Pyramid | 505 | 1.72 ± 0.22 | 0.235 | 161 |
|  | Bt Pyramid + SAI | 731 | 1.22 ± 0.14 | 0.045 | 163 |
| 12 | Non-RW Bt | 647 | 1.26 ± 0.19 | 0.271 | 25 |
|  | Non-RW Bt + SAI | 375 | 0.66 ± 0.17 | 0.000 | 59 |
|  | Bt Pyramid | 521 | 0.38 ± 0.08 | 0.000 | 31 |
|  | Bt Pyramid + SAI | 192 | 0.14 ± 0.05 | 0.000 | 38 |
| 15 | Non-RW Bt | 132 | 1.98 ± 0.17 | 0.352 | 118 |
|  | Non-RW Bt + SAI | 133 | 0.73 ± 0.08 | 0.000 | 149 |
|  | Bt Pyramid | 40 | 0.05 ± 0.01 | 0.000 | 167 |
|  | Bt Pyramid + SAI | 24 | 0.02 ± 0.01 | 0.000 | 153 |
| 16 | Non-RW Bt | 187 | 0.89 ± 0.09 | 0.000 | 205 |
|  | Non-RW Bt + SAI | 73 | 0.19 ± 0.03 | 0.000 | 224 |
|  | Bt Pyramid | 23 | 0.04 ± 0.01 | 0.000 | 195 |
|  | Bt Pyramid + SAI | 14 | 0.03 ± 0.01 | 0.000 | 202 |
| 18 | Non-RW Bt | 90 | 2.90 ± 0.06 | 0.790 | 149 |
|  | Non-RW Bt + SAI | 175 | 1.69 ± 0.15 | 0.539 | 181 |
|  | Bt Pyramid | 126 | 0.73 ± 0.18 | 0.297 | 178 |
|  | Bt Pyramid + SAI | 122 | 0.56 ± 0.12 | 0.108 | 190 |
| 19 | Non-RW Bt | 5 | 0.14 ± 0.03 | 0.000 | 262 |
|  | Non-RW Bt + SAI | 12 | 0.09 ± 0.01 | 0.000 | 270 |
|  | Bt Pyramid | 7 | 0.04 ± 0.01 | 0.000 | 222 |
|  | Bt Pyramid + SAI | 2 | 0.03 ± 0.01 | 0.000 | 255 |

Non-RW Bt: no corn rootworm traits; Bt pyramid: Cry3Bb1 + Gpp34Ab1/Tpp35Ab1; SAI: soil-applied insecticide; Total emergence from 4 single-plant emergence cages per treatment/site; NIS: 0-3 node injury scale, N=10 roots per treatment /site; Lodged plants: Mean proportion of plants leaning ≥ 45^o^ from stalk; Yield from 22.9 row-m per treatment.
