# Supplementary material for: Resistance management and integrated pest management insights from deployment of a Cry3Bb1+ Gpp34Ab1/Tpp35Ab1 pyramid in a resistant western corn rootworm landscape
Source: PLoS One. 2024 Mar 8;19(3):e0299483. doi: 10.1371/journal.pone.0299483 (PMC10923451; doi:10.1371/journal.pone.0299483)
Supplement: S6 Table — (A) Cry3Bb1, (B) Gpp34Ab1/Tpp35Ab1, (C) Cry3Bb1 + Gpp34Ab1/Tpp35Ab1 pyramid. Within hybrids, no significant differences in mean survival among colonies were documented (GLMM, binomial distribution; P>0.05). (DOCX) [file pone.0299483.s006.docx]

**S2.3 Table. Mean proportional survival (± SE) of susceptible lab control colonies in 2022 bioassays.** (A) Cry3Bb1, (B) Cry34/35Ab1, (C) Cry3Bb1 + Gpp34Ab1/Tpp35Ab1 pyramid. Within hybrids and years, no significant differences in mean survival among colonies were documented (GLMM, binomial distribution; *P*>0.05).
